# Prognostic Value of BIM Deletion in EGFR-Mutant NSCLC Patients Treated with EGFR-TKIs: A Meta-Analysis

**DOI:** 10.1155/2021/3621828

**Published:** 2021-10-13

**Authors:** Fangfang Lv, Liang Sun, Qiuping Yang, Zheng Pan, Yuhua Zhang

**Affiliations:** ^1^Department of Respiratory, Characteristic Medical Center of People's Armed Police Force, Tianjin 300162, China; ^2^Department of Immunology, Characteristic Medical Center of People's Armed Police Force, Tianjin 300162, China

## Abstract

**Background:**

Resistance to epidermal growth factor receptor tyrosine kinase inhibitors (EGFR-TKIs) is inevitable in EGFR-mutant non-small-cell lung cancer (NSCLC) patients. A germline 2903 bp deletion polymorphism of Bcl-2-like protein 11 (BIM) causes reduced expression of proapoptotic BH3-only BIM protein and blocks TKI-induced apoptosis of tumor cells. Yet the association between the deletion polymorphism and response to EGFR-TKI treatment remains inconsistent among clinical observations. Thus, we performed the present meta-analysis.

**Methods:**

Eligible studies were identified by searching PubMed, Embase, and ClinicalTrials.gov databases prior to March 31, 2021. Hazard ratios (HRs) and 95% confidence intervals (CIs) of progression-free survival (PFS) and overall survival (OS) and odds ratios (ORs) and 95% CIs of objective response rate (ORR) and disease control rate (DCR) were calculated by using a random effects model. Sensitivity, metaregression, and publication bias analyses were also performed.

**Results:**

A total of 20 datasets (3003 EGFR-mutant NSCLC patients receiving EGFR-TKIs from 18 studies) were included. There were 475 (15.8%) patients having the 2903-bp intron deletion of BIM and 2528 (84.2%) wild-type patients. BIM deletion predicted significantly shorter PFS (HR = 1.35, 95% CI: 1.10-1.64, *P* = 0.003) and a tendency toward an unfavorable OS (HR = 1.22, 95% CI: 0.99-1.50, *P* = 0.068). Patients with deletion polymorphism had lower ORR (OR = 0.60, 95% CI: 0.42-0.85, *P* = 0.004) and DCR (OR = 0.59, 95% CI: 0.38-0.90, *P* = 0.014) compared with those without deletion.

**Conclusion:**

BIM deletion polymorphism may confer resistance to EGFR-TKIs and can be used as a biomarker to predict treatment response to EGFR-TKIs in EGFR-mutant NSCLC patients from Asian populations.

## 1. Introduction

Lung cancer is the most prevalent malignant tumor with the highest mortality worldwide, which accounts for 11.6% of newly diagnosed cancers and 18.4% of cancer-related deaths in 2018 [1]. Non-small-cell lung cancer (NSCLC) accounts for 80~85% of lung cancer, and nearly 70% of NSCLC patients are diagnosed as having advanced disease [2, 3]. In recent years, molecular targeted therapy has shown great potentials in improving survivals, response, and quality of life and reducing adverse events and is recommended as the first-line therapy for advanced NSCLC with driven gene mutations according to National Comprehensive Cancer Network (NCCN) guidelines. Meanwhile, for those negative for driven gene mutations, chemoimmunotherapy or immune checkpoint inhibitors (ICIs) are currently the first-line treatment according to programmed cell death ligand 1 (PD-L1) score on tumor tissue.

Epidermal growth factor receptor (EGFR) mutations are important therapeutic targets for NSCLC, which can be found in 10% of Caucasian patients and 30~40% of Asian patients [4, 5]. In vitro experiments showed that NSCLC cell lines with EGFR mutations were hypersensitive to EGFR tyrosine kinase inhibitors (EGFR-TKIs) [6]. Numerous clinical trials demonstrated significantly prolonged progression-free survival (PFS), overall survival (OS), and higher objective response rate (ORR) in EGFR-mutant NSCLC patients receiving first-/second-generation EGFR-TKIs than those receiving chemotherapy [7–9]. In recent years, the third-generation EGFR-TKI osimertinib, which targets both EGFR-sensitive mutations and T790M-resistant mutation, has showed efficacy superior to other EGFR-TKIs in NSCLC patients with EGFR T790M-resistant mutation [10] or with EGFR-sensitive mutations [11, 12] and is widely adopted as first-line treatment in current clinical practice.

Despite the great efficacy of EGFR-TKIs, most patients ultimately have a relapse, indicating the development of drug resistance [13]. The acquired resistance to EGFR-TKIs may be mediated by secondary EFGR mutation (T790M for first-/second-generation TKIs, loss of T790M, and secondary C797S mutation for osimertinib), bypass pathway activation (MET/HER2 amplification, KRAS/BRAF/PIK3CA mutation, and RET/FGFR3/BRAF fusion), or small-cell lung cancer transformation [14, 15]. It is important to identify more biomarkers for TKI resistance and develop new treatment strategies to overcome the resistance.

Bcl-2-like protein 11 (BCL2L11 or BIM) belongs to the B-cell lymphoma-2 (BCL-2) family proteins that play key roles in regulating apoptosis of tumor cells [16]. BIM contains a BH3 domain which is essential for its proapoptosis activity. Costa et al. found that the upregulation of BIM correlated with gefitinib-induced apoptosis of EGFR-mutant lung cancer cells while knockdown of BIM blocked the apoptosis, indicating that BIM mediated TKI-induced apoptosis in lung cancer [17]. Furthermore, Ng et al. reported that a 2903 bp germline deletion polymorphism between exons 2 and 3 of BIM was associated with inferior response to EGFR-TKIs in EGFR-mutant NSCLC patients [18]. The deletion, causing incorrect splicing of exons 3 and 4, produces BIM-*γ* isoform that lacks the proapoptotic BH3 domain [18]. Therefore, this deletion polymorphism of BIM may modify the intrinsic resistance to EGFR-TKIs. Several clinical observations found that EGFR-mutant NSCLC patients carrying the 2903 bp deletion had shorter PFS and OS and were less responsive to EGFR-TKIs than those who did not have the deletion, suggesting that BIM deletion polymorphism may be an independent predictor for prognosis [18–20]. However, the predictive role of the polymorphism remains inconclusive since the results have not been replicated in recent studies [21–23]. Here, we performed a systematic review and meta-analysis to evaluate the association between the 2903 bp deletion polymorphism of BIM and treatment efficacy of EGFR-TKIs in EFGR-mutant NSCLC patients.

## 2. Methods

### 2.1. Literature Search and Selection Criteria

This meta-analysis was in accordance with the Preferred Reporting Items for Systematic Reviews and Meta-Analysis (PRISMA) statement. We searched articles investigating the association between BIM deletion polymorphism and efficacy of EGFR-TKIs in EGFR-mutant NSCLC patients in PubMed, Embase, and ClinicalTrials.gov databases prior to March 31, 2021. The following search terms were used: (BIM OR BCL2L11 OR Bcl-2-like protein 11) AND (lung cancer OR non-small cell lung cancer OR lung adenocarcinoma OR NSCLC). There was no language restriction. Additional eligible articles were obtained by reviewing the reference lists of relevant review and research articles.

Articles meeting the following criteria were considered eligible: (i) participants were NSCLC cases with EGFR activating mutations; (ii) patients were treated with EGFR-TKIs in any line; and (iii) survival outcomes (progression-free survival (PFS); overall survival (OS)) or treatment response (objective response rate (ORR); disease control rate (DCR)) for BIM deletion and wild-type groups were reported. Reviews, case reports, meeting abstracts, and those without sufficient data to estimate the effect size were discarded.

### 2.2. Data Extraction

We extracted the following information from each eligible article: first author, publication year, country, line of EGFR-TKIs, sample size, number of BIM deletion carriers, EGFR mutations, clinicopathological characteristics, smoking history, hazard ratio (HR) and corresponding 95% confidence interval (95% CI) for survival outcomes, BIM deletion distributions in patients with and without response, and so on. If an article did not report the HRs of survival outcomes, we extracted survival data from corresponding Kaplan-Meier curves using Engauge Digitizer software and estimated the HRs and 95% CI using the method introduced by Tierney et al. [24]. Data extraction was performed by two independent authors, and discrepancies were solved by full discussion.

### 2.3. Quality Assessment

The quality of included studies was assessed by using the Newcastle-Ottawa scale (NOS) [25]. NOS contains 3 domains with 8 items which are awarded with a total of 9 stars. Studies with 7 or more stars were considered of high quality.

### 2.4. Statistical Analysis

The between-study heterogeneity was assessed by *I*^2^ which indicated low (<25%), medium (25~50%), and high (>50%) heterogeneity, respectively. Considering the existence of heterogeneity, we applied a random effects model to all of the pooled analyses, which may generate more conservative results with wider confidence intervals than using a fixed effects model. The pooled HR for survival outcomes and odds ratio (OR) for response was calculated. In addition, we stratified the analysis by several moderators, including country (South Korea, China, and other countries), HR estimates (reported, estimated), and survival analysis (univariate, multivariate). Furthermore, to explore the other potential sources of heterogeneity, we performed metaregression analysis which allowed us to investigate the impact of several factors on pooled effect size. These factors included publication year, sample size, BIM deletion frequency, percent of adenocarcinoma cases, percent of first-line EGFR-TKI-treated patients, percent of ever-smoking patients, percent of male, percent of patients with Eastern Cooperative Oncology Group performance status (ECOG PS) ≥ 2, percent of stage IV or recurrent patients, and percent of patients harboring classic EGFR mutations (exon 19 deletions and exon 21 L858R). We also performed sensitivity analysis by excluding each study one at a time and pooling the others to assess the impact of a single study on the pooled effect size. Finally, we assessed the publication bias by a funnel plot and Egger's test. The meta-analysis was performed by using Stata 12.0 (Stata Corporation, TX, USA). The threshold of statistical significance was set as *P* < 0.05.

## 3. Results

### 3.1. Characteristics of Eligible Studies

**Figure 1:**
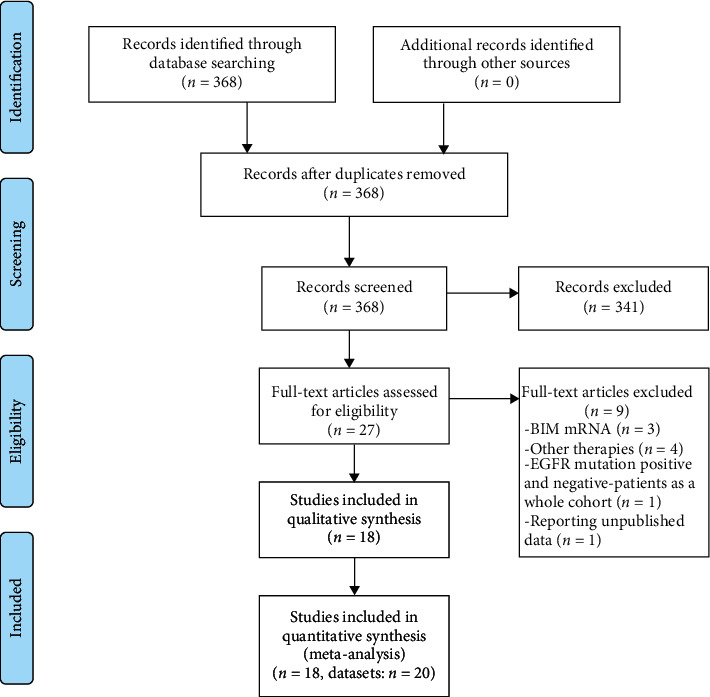
Flowchart of literature search.

Initially, a total of 368 articles were obtained by literature search, of which 341 were obviously not relevant and excluded. For the remaining candidate articles, 3 studied BIM mRNA expression [26–28], 4 reported the other therapies (resection, chemotherapy, radiotherapy, or crizotinib) [29–32], and 1 analyzed EGFR-mutant and EGFR-wild-type patients as a whole cohort [33]. Besides, 1 study reported 3 unpublished datasets that were requested from the other researchers [34]. Yet we could not determine whether they were duplicated with the other studies published later and had to discard them. Thus, these 9 studies were excluded. For 3 studies that also included EGFR-mutant and EGFR-wild-type patients simultaneously, we could extract the data from EGFR-mutant patients [19, 22, 35]. For two studies, each reported two unrelated cohorts [21, 23]. Finally, 20 datasets from 18 studies [18–23, 35–46] exploring the association between BIM deletion polymorphism and EGFR-TKI efficacy in EGFR-mutant NSCLC patients were included in our meta-analysis (Figure 1).

Among the 3003 EGFR-mutant NSCLC patients included in our meta-analysis, 475 had the 2903 bp intron deletion of BIM. The frequencies of BIM deletion ranged between 9.6% and 26.4%. All of the studies enrolled stage III, IV, or recurrent patients except one including patients of all stages [35] and one not reporting stages [46]. The first-generation TKIs including gefitinib and erlotinib were the most frequently used while afatinib and icotinib were also given to some patients. Notably, Li et al. enrolled patients positive for EGFR T790M-resistant mutation and treated with osimertinib [45]. Only one study was prospectively designed [19] while the others were all retrospective. For survival outcomes, all of the 20 datasets reported PFS and 13 reported OS. For response, 14 and 12 datasets reported ORR and DCR, respectively. All studies were awarded with 7 or more stars and were considered to be of high quality. The characteristics of included studies for survival and response outcomes are shown in Tables 1 and 2, respectively.

### 3.2. BIM Deletion Predicted Unfavorable PFS and OS

**Figure 2:**
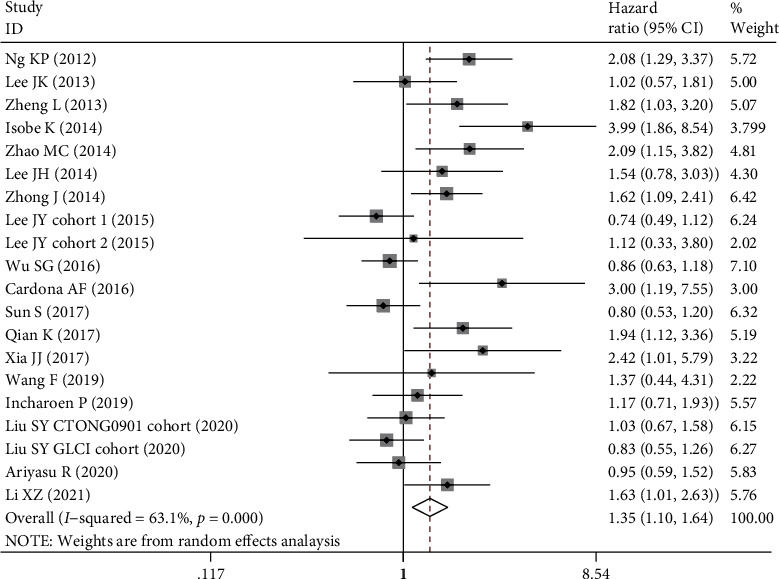
Forest plot of BIM deletion polymorphism associated with progression-free survival in EGFR-mutant NSCLC patients treated with EGFR-TKIs.

Twenty datasets comprising 3003 patients explored the predictive role of BIM deletion in PFS (Table 3). Meta-analysis demonstrated that BIM deletion carriers had significantly shorter PFS than wide-type carriers (HR = 1.35, 95% CI: 1.10-1.64, *P* = 0.003, Figure 2). Even if two outliers with HR ≥ 3.00 were excluded [42, 44], the association was still significant (HR = 1.24, 95% CI 1.04-1.49, *P* = 0.020), indicating that the result was robust. Stratified analysis showed that BIM deletion predicted unfavorable PFS in the Chinese population (HR = 1.32, 95% CI 1.05-1.66, *P* = 0.019) and the other populations (HR = 1.80, 95% CI 1.09-2.99, *P* = 0.022) but not in the South Korean population (HR = 0.84, 95% CI 0.61-1.17, *P* = 0.310).

**Figure 3:**
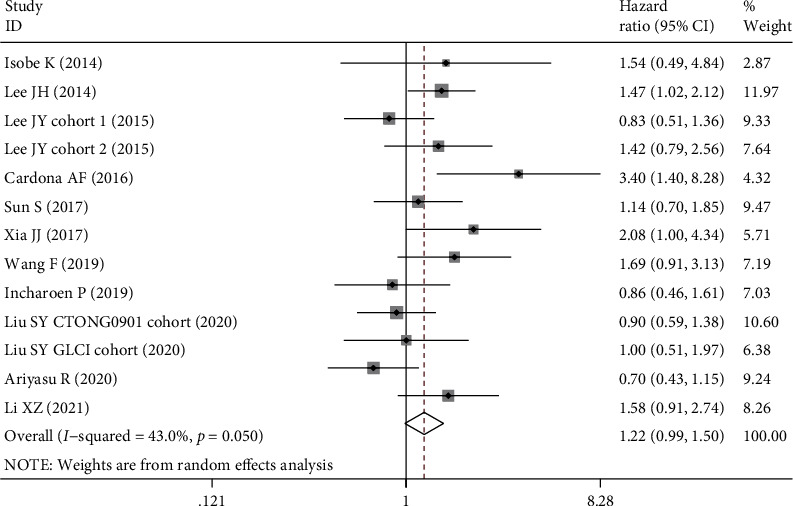
Forest plot of BIM deletion polymorphism associated with overall survival in EGFR-mutant NSCLC patients treated with EGFR-TKIs.

The association between BIM deletion and OS was evaluated in 13 datasets (Table 4) with 1830 EGFR-mutant NSCLC patients. Our analysis indicated that BIM deletion was related to a shorter OS, but the association was not significant (HR = 1.22, 95% CI: 0.99-1.50, *P* = 0.068, Figure 3). Subgroup analysis in the Chinese population rather than the other populations demonstrated a significant association between BIM deletion and OS (HR = 1.30, 95% CI: 1.06-1.60, *P* = 0.013).

### 3.3. BIM Deletion Predicted Lower Response Rates

**Figure 4:**
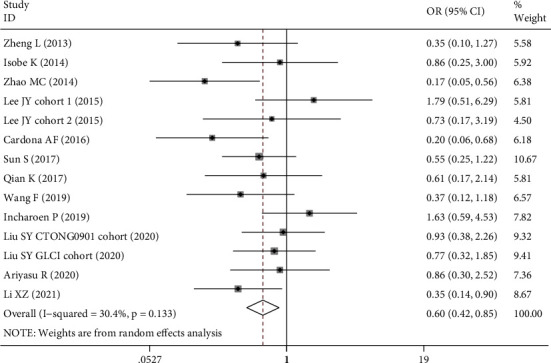
Forest plot of BIM deletion polymorphism associated with objective response rate in EGFR-mutant NSCLC patients treated with EGFR-TKIs.

**Figure 5:**
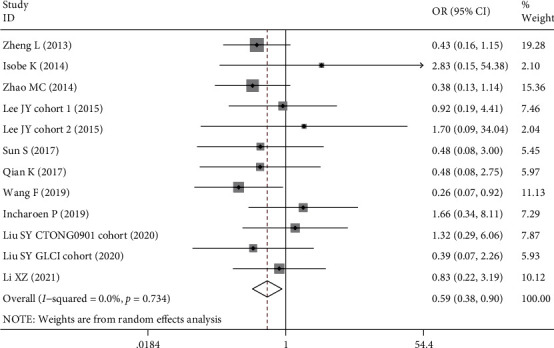
Forest plot of BIM deletion polymorphism associated with disease control rate in EGFR-mutant NSCLC patients treated with EGFR-TKIs.

The association between BIM deletion and treatment response to EGFR-TKIs was also analyzed (Table 5). Our results demonstrated that BIM deletion polymorphism was associated with lower objective response rate (OR = 0.60, 95% CI: 0.42-0.85, *P* = 0.004, Figure 4) and disease control rate (OR = 0.59, 95% CI: 0.38-0.90, *P* = 0.014, Figure 5). If two outliers with OR ≤ 0.20 were excluded [36, 44], the association for ORR was still significant (OR = 0.71, 95% CI 0.52-0.96, *P* = 0.025, *I*^2^ = 0), indicating that the result was robust. The significant associations were found in the subgroup of the Chinese population that carriers of BIM deletion were less likely to achieve objective response (OR = 0.50, 95% CI 0.35-0.71, *P* < 0.001) or disease control (OR = 0.48, 95% CI 0.30-0.77, *P* = 0.002).

### 3.4. Metaregression Analysis, Sensitivity Analysis, and Publication Bias

**Figure 6:**
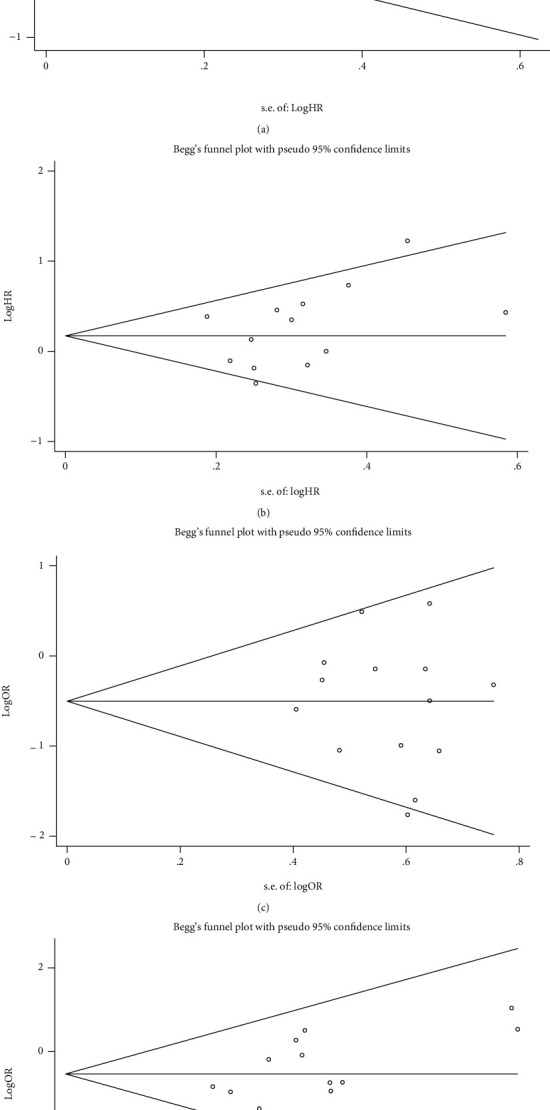
Funnel plots for progression-free survival (a), overall survival (b), objective response rate (c), and disease control rate (d).

The results of metaregression analysis are shown in Supplementary Table 1. Sample size was suggested as a modulator for the effect size of PFS at borderline significance (*P* = 0.040). The other factors were not the source of between-study heterogeneity according to metaregression analysis. Yet subgroup analysis stratified by country showed that there was low between-study heterogeneity in studies from China. Sensitivity analysis showed that none of the included studies had significant impact on the pooled effect size. The funnel plot for PFS was obviously asymmetric which indicated potential publication bias (Egger's test, *P* = 0.009), while the funnel plots for OS, ORR, and DCR were all symmetric (Figure 6).

## 4. Discussion

BIM encodes a BH3-only protein crucial for BCL-2-induced apoptosis of tumor cells. The 2903 bp deletion polymorphism leads to significantly reduced expression or absence of functional protein containing the BH3 domain and interrupts the apoptosis process of EGFR-mutant tumor cells induced by EGFR-TKIs [18]. The present meta-analysis of clinical observations demonstrated that the presence of deletion was significantly associated with shorter PFS, lower ORR and DCR, and a tendency toward an unfavorable OS. In particular in the Chinese population, BIM deletion polymorphism predicted inferior survival and treatment response. Therefore, the BIM deletion polymorphism confers resistance to EGFR-TKIs and can be used as a predictor of treatment efficacy and prognosis of EGFR-mutant NSCLS patients treated with EGFR-TKIs from Asian populations.

In addition to the deletion polymorphism, the expression levels of BIM mRNA were also associated with responsiveness to EGFR-TKIs. NSCLC patients with high expression levels had significantly prolonged PFS and OS than those with low/intermediate levels [26–28]. Either the polymorphism or the mRNA expression may confer resistance to TKIs through reducing the product of proapoptotic BH3-containing BIM protein. One possible strategy to overcome the resistance and restore the response to TKIs is adding BH3-mimetic drug or histone deacetylase (HDAC) inhibition. In vitro experiments revealed that the addition of BH3-mimetic drug ABT-737 with imatinib enhanced the TKI-induced apoptosis and cell death in deletion-containing cells [18, 47]. The other studies demonstrated that the HDAC inhibitor vorinostat could circumvent TKI resistance in EGFR-mutant NSCLC cell lines harboring BIM deletion polymorphism [48, 49]. A phase I study evaluated the effect of vorinostat plus gefitinib in BIM deletion/EGFR mutation double-positive NSCLC patients [50]. The median PFS was 5.2 months, and DCR at 6 weeks was 83.3% [50]. However, the therapeutic effect of the combination of BH3-mimetic drugs or HDAC inhibitors with EGFR-TKIs in patients developing BIM deletion polymorphism-mediated resistance needs to be validated by more clinical trials.

Besides the proapoptosis activity, BIM may play a crucial role in tissue vascularization. Bim(-/-) retinal endothelial cells have increased proliferation, migration, and vascular endothelial growth factor (VEGF) expression [51]. In lung endothelial cells, lack of Bim expression increased migration [52]. The conditional lack of Bim in mice attenuates hyaloid vessel regression and promotes retinal vascular remodeling [53]. These findings suggest a relevance between BIM and angiogenesis. Previous clinical trials demonstrated that erlotinib combined with antiangiogenic drugs, such as ramucirumab and bevacizumab, increased PFS but not treatment response in advanced NSCLC [54, 55]. A recent retrospective study analyzed the clinical efficacy of EGFR-TKI plus bevacizumab (VEGF inhibitor) versus EGRK-TKI alone in advanced NSCLC patients with EFGR mutations and BIM deletion and found that the addition of bevacizumab resulted in significantly higher ORR, longer PFS, and a tendency toward a favorable OS [56]. It seems that the combination of EGFR-TKIs and antiangiogenic agents may be more effective in patients with BIM deletion polymorphism, which needs comparison to patients negative for the polymorphism in the future. Nonetheless, this provides a possible strategy to improve treatment response to EFGR-TKIs by adding VEGR inhibitors in this group of patients.

Apart from EGFR-TKIs, BIM deletion polymorphism may also mediate the resistance to crizotinib, the first-generation anaplastic lymphoma kinase (ALK) TKI. ALK fusion-positive NSCLC patients with BIM deletions had a significantly shorter PFS and lower ORR than those without the polymorphism [57]. However, Lin et al. did not found a positive association between the polymorphism and PFS or OS [29]. More evidence needs to be collected to determine the predictive role of BIM deletion polymorphism in ALK TKI-treated NSCLC patients.

Despite the prognostic value, the deletion polymorphism can only be used as a biomarker in the Asian population since the polymorphism is not found in Caucasians or Africans [18]. Studies included in our meta-analysis were mainly conducted in Singapore, China, Japan, South Korea, and Thailand, while only one was in Columbia with Hispanic patients [44]. The prevalence of deletion polymorphism varied between 9.6% and 26.4% in these studies, and the mean frequency was 15.8% (475/3003).

Compared with previous meta-analyses [39, 58–61], the present study has several strengths. Firstly, our study exclusively included EGFR-mutant patients to keep individual homogeneity while several previous meta-analyses did not carefully discriminate EGFR mutation status [39, 58] when screening eligible studies. Secondly, our study had the largest sample size (3003 EGFR-mutant NSCLC patients) compared to the previous ones by adding more recent publications. Thirdly, we applied a random effects model to all the pooled analyses regardless of between-study heterogeneity, considering that potential sources of heterogeneity, including genetic background, clinicopathological features, or the other possible confounders, may vary among included studies. This generates more conservative results and wider confidence intervals compared with using a fixed effects model. In the present study, we found that BIM deletion polymorphism was significantly associated with clinical and survival outcomes in EGFR-mutant NSCLC patients, especially in the Chinese population which has not been revealed by previous meta-analyses.

However, our study has several limitations. Firstly, almost all of the included studies were retrospectively designed, which unavoidably introduced bias. More prospective studies are needed in the future. Secondly, most of included studies were investigating the first-generation EGFR-TKIs, and only one study for osimertinib, a third-generation EGFR-TKI with widespread utilization in current clinical practice, was available for our analysis. BIM deletion was associated with shorter PFS and lower ORR in EGFR T790M NSCLC patients treated with osimertinib [45], which needs validation in more populations. Thirdly, most of the studies were performed in Asian populations since the BIM deletion was rare in Caucasian populations, which limited the application of our results.

## 5. Conclusions

In conclusion, the 2903 bp deletion polymorphism of BIM is associated with poor response to EGFR-TKIs, primarily the first-generation inhibitors, in EGFR-mutant NSCLC patients. BIM deletion polymorphism can be used as a prognostic marker in this group of patients from Asian populations.

## Figures and Tables

**Table 1:** Characteristics of included studies for survival analysis.

Author	Year	Country	BIM deletion/total	Line of EGFR-TKIs	NSCLC stage	Median PFS, months (deletion/WT)	Median OS, months (deletion/WT)	NOS stars
Ng KP	2012	Singapore	26/141	First or more	III, IV, recurrent	6.6/11.9	NR	7
Lee JK	2013	South Korea	21/193	First or more	IIIB, IV, recurrent	11.9/11.3	NR	8
Zheng L	2013	China	21/123	Second or more	IIIB, IV	3.5/6.0	NR	8
Isobe K	2014	Japan	13/70	First or more	IV, recurrent	7.6/17.8	39.2/45.4	7
Zhao MC	2014	China	16/166	First or more	IIIB, IV	4.7/11.0	NR	9
Lee JH	2014	China	16/80	First	IIIB, IV	7.4/9.4	18.3/24.9	7
Zhong J	2014	China	24/159	First or more	I-IV	7.3/9.5	NR	7
Lee JY cohort 1	2015	South Korea	32/205	First or more	IIIB, IV, recurrent	11.9/10.9	31.2/30.3	7
Lee JY cohort 2	2015	South Korea	10/69	First or more	IIIB, IV, recurrent	11.6/9.7	NR	7
Wu SG	2016	China	52/327	NR	IV	10.5/8.5	NR	8
Cardona AF	2016	Columbia	14/89	First	IIIA, IIIB, IV	10.8/21.7	15.5/34.0	7
Sun S	2017	China	37/140	First or more	III, IV	21/17	34/33	7
Qian K	2017	China	14/85	First	IIIB, IV	7.1/12.8	NR	7
Xia JJ	2017	China	43/245	First or more	IIIB, IV, recurrent	22/38	24/39	8
Wang F	2019	China	18/169	First or more	IIIB, IV, recurrent	NR	NR	9
Incharoen P	2019	Thailand	20/97	First or more	IV, recurrent	8.6/8.9	25.8/28.9	8
Liu SY CTONG0901	2020	China	22/194	First or more	IIIB, IV	10.5/11.2	20.5/20.5	7
Liu SY GLCI	2020	China	24/141	First or more	IIIB, IB	10.1/11.6	58.5/45.0	7
Ariyasu R	2020	Japan	27/167	First or more	NR	10.3/10.4	38.4/31.6	8
Li XZ^#^	2021	China	25/143	Second or more	IIIB, IV	8.3/10.5	15.9/25.2	8

^#^All patients were positive for EGFR T790M and received osimertinib. EGFR-TKIs: epidermal growth factor receptor tyrosine kinase inhibitors; NSCLC: non-small-cell lung cancer; PFS: progression-free survival; OS: overall survival; WT: wild-type BIM; NOS: Newcastle-Ottawa scale; NR: not reported.

**Table 2:** Characteristics of included studies for treatment response to EGFR-TKIs.

Author	Year	ORR^#^	Non-ORR^#^	DCR^&^	Non-DCR^&^	Response criteria
Zheng L	2013	3/33	18/69	12/77	9/25	RECIST v1.1
Isobe K	2014	8/37	5/20	13/52	0/5	NR
Zhao MC	2014	4/99	12/51	10/122	6/28	RECIST v1.1
Lee JY cohort 1	2015	29/146	3/27	30/163	2/10	RECIST v1.1
Lee JY cohort 2	2015	7/45	3/14	10/55	0/4	RECIST v1.1
Cardona AF	2016	5/55	9/20	NR	NR	NR
Sun S	2017	16/63	17/37	31/97	2/3	RECIST v1.1
Qian K	2017	4/27	10/41	12/63	2/5	RECIST
Wang F	2019	4/64	14/83	14/137	4/10	RECIST v1.0
Incharoen P	2019	13/41	7/36	18/65	2/12	RECIST v1.1
Liu SY CTONG0901	2020	12/97	10/75	20/152	2/20	NR
Liu SY GLCI	2020	13/71	11/46	22/113	2/4	NR
Ariyasu R	2020	22/117	5/23	NR	NR	RECIST v1.1
Li XZ	2021	7/62	18/56	22/106	3/12	RECIST v1.1

^#^Number of patients who achieved objective response (ORR) and who did not achieve objective response (non-ORR) in the BIM deletion group/wild-type group, respectively. ^&^Number of patients who achieved disease control (DCR) and who did not achieve disease control (non-DCR) in the BIM deletion group/wild-type group, respectively. ORR: objective response rate; DCR: disease control rate; RECIST: Response Evaluation Criteria in Solid Tumors; NR: not reported.

**Table 3:** Meta-analysis of BIM deletion polymorphism associated with PFS.

Subgroup	No. of studies	BIM deletion/total	*I* ^2^ (%)	Pooled HR	95% CI	*P*
Overall	20	475/3003	63.1	1.35	1.10-1.64	0.003
Country						
South Korea	3	63/467	0	0.84	0.61-1.17	0.310
China	12	312/1972	58.9	1.32	1.05-1.66	0.019
Others	5	100/564	72.8	1.80	1.09-2.99	0.022
HR estimates						
Reported	14	365/2275	69.3	1.39	1.07-1.81	0.013
Estimated	6	110/728	44.6	1.28	0.95-1.71	0.100
Survival analysis						
Univariate	13	333/2104	44.8	1.10	0.91-1.33	0.331
Multivariate	7	142/899	54.8	1.89	1.37-2.62	<0.001

PFS: progression-free survival; HR: hazard ratio. All HRs were pooled by a random effects model.

**Table 4:** Meta-analysis of BIM deletion polymorphism associated with OS.

Subgroup	No. of studies	BIM deletion/total	*I* ^2^ (%)	Pooled HR	95% CI	*P*
Overall	13	303/1830	43.0	1.22	0.99-1.50	0.068
Country						
China	7	187/1133	12.5	1.30	1.06-1.60	0.013
Others	6	116/697	58.1	1.12	0.75-1.69	0.579
HR estimates						
Reported	8	216/1255	59.6	1.25	0.90-1.74	0.178
Estimated	5	87/575	0	1.21	0.96-1.53	0.101
Survival analysis						
Univariate	11	262/1574	11.0	1.22	1.02-1.45	0.029
Multivariate	2	41/256	89.2	1.47	0.31-6.92	0.622

OS: overall survival; HR: hazard ratio. All HRs were pooled by a random effects model.

**Table 5:** Meta-analysis of BIM deletion polymorphism associated with response to EGFR-TKIs.

Response	No. of studies	BIM deletion/total	*I* ^2^ (%)	Pooled OR	95% CI	*P*
ORR						
Overall	14	289/1844	30.4	0.60	0.42-0.85	0.004
China	8	173/1147	1.9	0.50	0.35-0.71	<0.001
Others	6	116/697	40.4	0.85	0.45-1.59	0.605
DCR						
Overall	12	248/1588	0	0.59	0.38-0.90	0.014
China	8	173/1147	0	0.48	0.30-0.77	0.002
Others	4	75/441	0	1.40	0.52-3.75	0.504

ORR: objective response rate; DCR: disease control rate; OR: odds ratio.

## Data Availability

The data used to support the findings of this study are available from the corresponding author upon request.

## Supplementary Materials

Supplementary 1 Supplementary File 1. Supplementary Table 1: results of metaregression analysis.

Supplementary 2 Supplementary File 2: PRISMA 2009 checklist.
