# Supplementary material for: Prognostic Value of BIM Deletion in EGFR-Mutant NSCLC Patients Treated with EGFR-TKIs: A Meta-Analysis
Source: Biomed Res Int. 2021 Oct 13;2021:3621828. doi: 10.1155/2021/3621828 (PMC8551980; doi:10.1155/2021/3621828)
Supplement: Supplementary 1 — Supplementary File 1. Supplementary Table 1: results of metaregression analysis. [file 3621828.f1.docx]

Supplementary table 1 Results of meta-regression analysis

| Outcome | Moderator | Co-efficient | SE | t | P |
| --- | --- | --- | --- | --- | --- |
| PFS |  |  |  |  |  |
|  | Publication year | -0.059 | 0.035 | -1.66 | 0.114 |
|  | Sample size | -0.003 | -0.001 | -2.21 | 0.004 |
|  | % BIM deletion | -0.871 | 2.741 | -0.32 | 0.754 |
|  | % Adenocarcinoma | -2.820 | 1.931 | -1.45 | 0.164 |
|  | % First-line treatment | 0.046 | 0.354 | 0.13 | 0.899 |
|  | % Ever smoking | 1.480 | 1.330 | 1.11 | 0.281 |
|  | % Male | -0.904 | 1.697 | -0.53 | 0.601 |
|  | % ECOG PS ≥2 | 1.913 | 1.334 | 1.43 | 0.175 |
|  | % Stage IV or recurrent NSCLC | -1.063 | 1.225 | -0.87 | 0.400 |
|  | % Classic EGFR mutations^#^ | 0.366 | 3.183 | 0.11 | 0.910 |
| OS |  |  |  |  |  |
|  | Publication year | -0.048 | 0.045 | -1.07 | 0.308 |
|  | Sample size | -0.002 | 0.002 | -0.92 | 0.375 |
|  | % BIM deletion | -0.382 | 2.855 | -0.12 | 0.896 |
|  | % Adenocarcinoma | -1.266 | 4.691 | -0.27 | 0.793 |
|  | % First-line treatment | 0.096 | 0.439 | 0.22 | 0.931 |
|  | % Ever smoking | 0.353 | 1.547 | 0.23 | 0.824 |
|  | % Male | -0.617 | 1.870 | -0.33 | 0.748 |
|  | % ECOG PS ≥2 | 2.280 | 1.298 | 1.76 | 0.113 |
|  | % Stage IV or recurrent NSCLC | -2.565 | 3.079 | -0.83 | 0.429 |
|  | % Classic EGFR mutations^#^ | -2.231 | 3.790 | -0.59 | 0.569 |
| ORR |  |  |  |  |  |
|  | Publication year | 0.062 | 0.071 | 0.86 | 0.405 |
|  | Sample size | 0.002 | 0.005 | 0.33 | 0.746 |
|  | % BIM deletion | 4.387 | 4.449 | 0.99 | 0.344 |
|  | % Adenocarcinoma | 5.104 | 2.938 | 1.74 | 0.110 |
|  | % First-line EGFR-TKIs | 0.345 | 0.587 | 0.59 | 0.570 |
|  | % Ever smoking | -1.878 | 2.194 | -0.86 | 0.409 |
|  | % Male | -2.995 | 2.439 | -1.23 | 0.243 |
|  | % ECOG PS ≥2 | -2.183 | 2.016 | -1.08 | 0.304 |
|  | % Stage IV or recurrent NSCLC | 1.345 | 1.953 | 0.69 | 0.507 |
|  | % Classic EGFR mutations^#^ | -5.539 | 6.146 | -0.90 | 0.389 |

PFS: progression-free survival; OS: overall survival; ORR: objective response rate; ECOG PS: Eastern Cooperative Oncology Group

performance status; ^#^ Exon 19 deletion and exon 21 L858R.
